# Ultracompact Programmable Silicon Photonics Using Layers of Low-Loss Phase-Change Material Sb_2_Se_3_ of Increasing Thickness

**DOI:** 10.1021/acsphotonics.4c01789

**Published:** 2025-03-07

**Authors:** Sophie Blundell, Thomas W. Radford, Idris A. Ajia, Daniel Lawson, Xingzhao Yan, Mehdi Banakar, David J. Thomson, Ioannis Zeimpekis, Otto L. Muskens

**Affiliations:** † Optoelectronics Research Centre, University of Southampton, Southampton SO17 1BJ, U.K.; ‡ School of Physics and Astronomy, University of Southampton, Southampton SO17 1BJ, U.K.; § Electronics and Computer Science, University of Southampton, Southampton SO17 1BJ, U.K.

**Keywords:** silicon photonics, programmable photonic devices, phase change, Sb_2_Se_3_

## Abstract

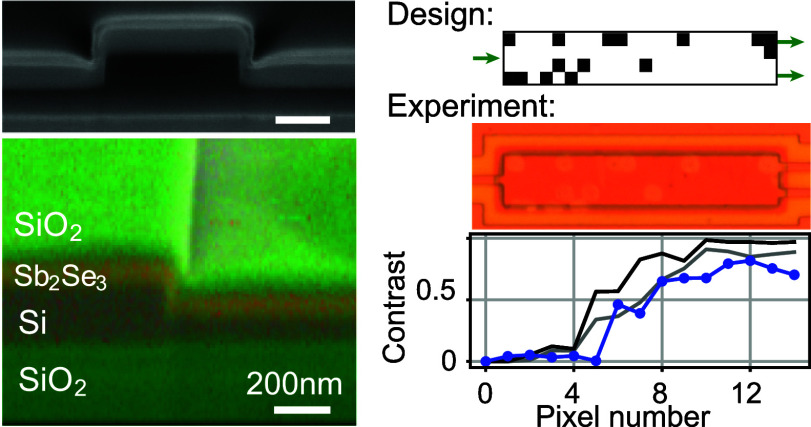

High-performance programmable silicon photonic circuits are considered to be a critical part of next-generation architectures for optical processing, photonic quantum circuits, and neural networks. Low-loss optical phase-change materials (PCMs) offer a promising route toward nonvolatile free-form control of light. Here, we exploit the direct-write digital patterning of waveguides using layers of the PCM Sb_2_Se_3_ with thickness values from 20 to 100 nm, demonstrating the scaling of induced optical phase shift with thickness and the ability to strongly increase the effect per pixel for thicker layers. We exploit the excellent refractive index matching between Sb_2_Se_3_ and silicon to achieve a low-loss hybrid platform for programmable photonics. A 5-fold reduction in the modulation length of a Mach–Zehnder interferometer is achieved with increasing thickness compared to the 20 nm thin-film Sb_2_Se_3_ devices, which decreased to 5 μm in this work. Application of the thicker PCM layers in direct-write digital programming of a multimode interferometer shows a corresponding 3-fold reduction of the number of programmed pixels to below 10 pixels per device. The demonstrated scaling of performance with Sb_2_Se_3_ layer thickness is important for establishing the optimum working range for hybrid silicon-Sb_2_Se_3_ devices and holds promise for achieving ultracompact, programmable photonic circuits.

## Introduction

The area of photonics exploiting phase-change materials (PCMs) for reconfiguring and programming functional devices has seen an enormous increase in activity, spurred by the recent availability of new materials that can be integrated into technologies such as integrated photonics and metamaterials.^1−4^ The capability to write and reset optical functionality after device fabrication is critical for a wide range of emerging applications such as quantum photonics,^5^ optical neural networks,^6,7^ microwave photonics,^8^ beamforming and lidar,^9^ and optical data processing.^10,11^ Compared to fully programmable mesh structures which require a continuous energy input to maintain their state,^10^ nonvolatile PCMs only require energy input for their initialization and reset operations, a distinct advantage compared to existing technologies.^12,13^ Programmable elements may also be used for postfabrication diversification of photonic chips, to mitigate the lengthy cycle of application-specific silicon photonics device design and fabrication.^14^

Applications of nonvolatile tunable and reconfigurable photonics depend critically on the development of new optical PCMs that can provide control over the optical phase of light without introducing large losses^2^ while maintaining a high endurance for repeated cycling between the material states.^15^ Following the introduction of the low-loss PCM antimony selenide (Sb_2_Se_3_) for silicon photonics,^16^ devices exploiting thin Sb_2_Se_3_ films on top of photonic waveguides have seen a highly successful development in recent years by an increasing number of researchers worldwide, which has included demonstrations of direct-write optical programming^17,18^ as well as electrical switching using integrated pn-junctions^19,20^ and graphene microheaters.^21^ The real-world impact of these materials is evidenced by their rapid integration into CMOS platforms compatible with commercial semiconductor foundry technology.^22,23^ A wide range of thicknesses have been reported in the literature for other PCM families integrated in platforms such as metamaterials, hybrid plasmonic devices, and photonic integrated circuits for optical phase control.^1−4,12^ Recent research addressing the integration of the new low-loss family of PCMs Sb_2_Se_3_ and Sb_2_S_3_ with silicon photonics has focused on thin layers of around 20 nm thickness. The dependence of the performance of programmable silicon photonics on the PCM layer thickness for these materials has remained largely unexplored.

The low-loss optical PCM Sb_2_Se_3_ is of particular interest for silicon photonics because of its refractive index which is closely matched to silicon, allowing for seamless integration; furthermore, it exhibits a sizable switching contrast between the amorphous and crystalline states of Δ*n* = 0.77 and shows very low intrinsic material losses around the telecommunication band at 1550 nm wavelength.^16^ A reversible change in optical phase exceeding π was demonstrated through direct optical writing of a 23 nm thick slab of Sb_2_Se_3_ deposited on top of a 220 nm SOI rib waveguide located in one of the arms of an asymmetric Mach–Zehnder interferometer (MZI) device.^17^ With this low thickness of Sb_2_Se_3_, the optical phase shift was found to be around 0.04π per μm, resulting in a device length *L*_π_ of around 25 μm. Subsequent works reported similar magnitudes of the effect exploiting electrical switching.^19−21^ While these very thin films of the low-loss PCM Sb_2_Se_3_ offer good performance for the optical phase control of silicon photonic devices, increasing the optical phase shift per device length would be of interest to achieve more compact device geometries. Thicker PCM layers could provide a shorter device length by increasing the switching contrast of the mode index between the material states; however, this has to be traded off against increased losses by absorption, scattering, and multimode contributions. Recent studies on continuous Sb_2_Se_3_ films show that reversible switching over more than a million cycles can be achieved for PCM layer thicknesses of up to 200 nm.^24,25^

In this work, we investigate the role of the thickness of low-loss PCM Sb_2_Se_3_ on the performance of programmable silicon-on-insulator (SOI) photonic devices. We exploit the excellent matching of the refractive index of Sb_2_Se_3_ to silicon to ensure good mode overlap and low insertion losses in the amorphous phase. Integration of the low-loss optical PCM Sb_2_Se_3_ in a silicon photonic device provides a method for controlling the optical phase without introducing very large transmission losses. The main figure of merit (FOM) for hybrid silicon-PCM devices is the induced optical phase shift between the switched states, Δϕ, normalized against the device loss, α, caused by scattering or absorption in the waveguide. In the devices under study, α is governed primarily by losses in the crystalline state.

The first part of this work is aimed at determining this FOM of hybrid silicon-Sb_2_Se_3_ devices with increasing Sb_2_Se_3_ thickness. The device design and fabrication are introduced, followed by optical studies of device losses α in straight waveguides (SWGs). The induced optical phase shift Δϕ is studied in MZI devices to arrive at the device switching FOM Δϕ/α. The second part of the study investigates the capability of thicker PCM layers in the digital patterning of multimode interference devices (MMIs). Digital patterning of weak perturbations, as described by us in previous works^17,26,27^ is distinct from topological inverse design, which relies on the precise shaping of complex patterns with ultrafine features.^28,29^ Digital patterning is typically achieved over a much coarser grid of perturbation coordinates, making use of multiple scattering and mode mixing at the individual perturbations.^26^ Here, we investigate how the number of pixels required to control a multimode device scales with PCM thickness. An optimal working point in the number of pixels required to switch the device, achieving a high level of control over the output state while minimizing programming effort, is of interest for achieving a platform for digital programmable photonics.^17,27^

## Results and Discussion

### Design and Fabrication of Hybrid Silicon-Sb_2_Se_3_ Devices

Silicon photonic devices were fabricated using a 220 nm SOI platform,^30^ as explained in the Methods section. Photonic rib waveguides of 120 nm thickness were covered with patches of low-loss PCM Sb_2_Se_3_. A 50 nm thin SiO_2_ cladding was used as a protection layer on top of the Sb_2_Se_3_ PCM.

**Figure 1:**
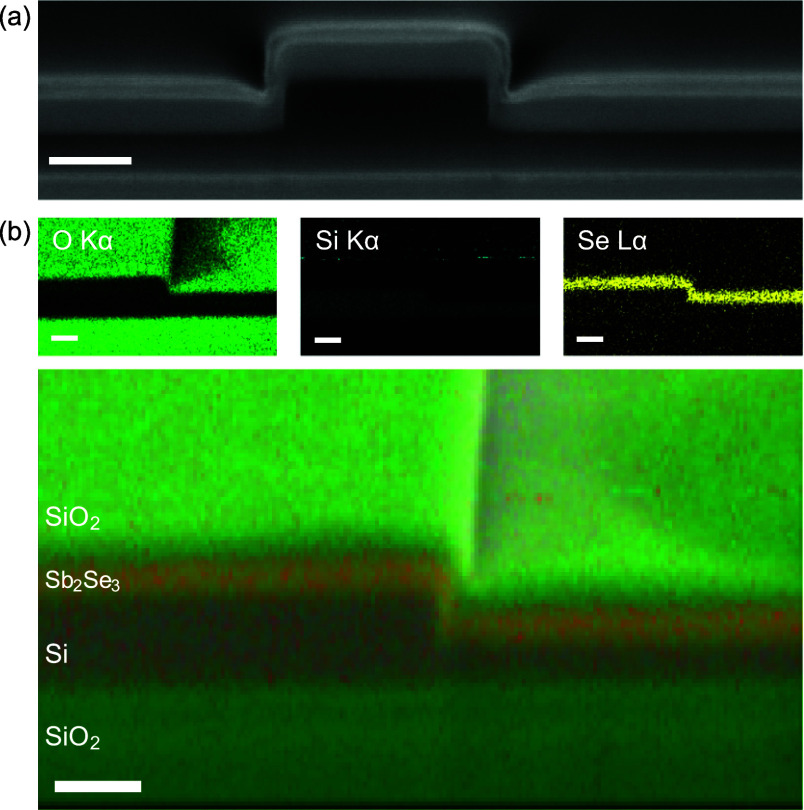
(a) Cross-sectional SEM image of the silicon waveguide with an Sb_2_Se_3_ layer of 100 nm thickness and 20 nm SiO_2_ cladding. (b) EDS analysis of the MMI device showing oxygen (O Kα), silicon (Si Kα), and selenium (Se Lα) signatures in separate panels and in the SEM overlay (large panel). All scale bars in (a, b) are 200 nm.

Figure 1a shows a scanning electron microscopy (SEM) cross section of a silicon rib waveguide covered with an Sb_2_Se_3_ layer of 100 nm thickness. The SEM allows identification of different layers in the stack through their contrast in the backscattered electron image, with the Sb_2_Se_3_ layer being the light-gray region on top of the darker SOI waveguide. More specific elemental information is obtained from energy-dispersive spectroscopy (EDS) at 2 kV, as shown in Figure 1b for a region of an MMI device. The EDS map clearly identifies the Sb_2_Se_3_ layer through the Se Lα contribution. We found good agreement between the designed and fabricated thickness of the Sb_2_Se_3_ layer. The aim of an Ar-plasma pretreatment in the process flow was to achieve a partial embedding of the PCM into the waveguide; however, the cross section reveals that the Ar-plasma only marginally affected the Si thickness and the Sb_2_Se_3_ layer sits on top of the waveguide. A discussion of the device geometry, with numerical simulations of the effective index contrast for different embedding depths, is presented in more detail in the Supporting Information Section S1. The difference between embedded and nonembedded PCM in the optical switching response is found to be modest and equivalent to a variation of around 20 nm of the PCM thickness itself.

**Figure 2:**
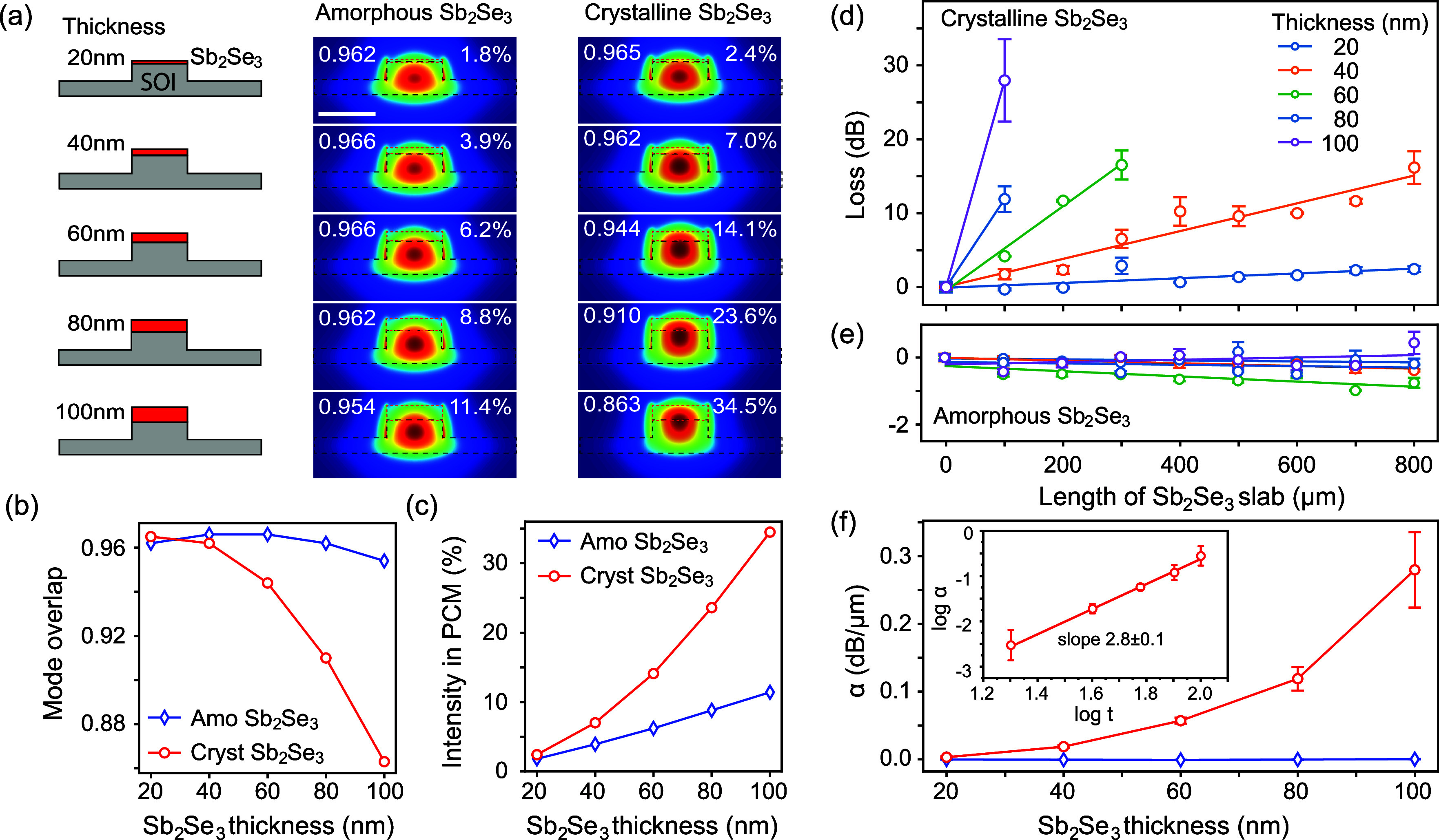
(a–c) Simulated mode profiles (a) for five different Sb_2_Se_3_ thickness values from 20 to 100 nm and for crystalline and amorphous Sb_2_Se_3_ states. Scale bar is 500 nm. Numbers in top left of the panels indicate mode overlap with the fundamental 220 nm SOI mode and are plotted in (b) against Sb_2_Se_3_ thickness. Numbers in top right indicate the percentage of mode intensity inside the Sb_2_Se_3_ layer and are plotted in (c) against Sb_2_Se_3_ thickness. (d, e) Experimental insertion loss of SWGs with Sb_2_Se_3_ slabs of varying lengths and for Sb_2_Se_3_ thickness from 20 to 100 nm, for the crystalline state (d) and the amorphous state (e), normalized to the SWG without PCM. (f) Propagation loss α in dB/μm against Sb_2_Se_3_ layer thickness for the amorphous state (diamond, blue) and the crystalline state (circle, red).

Figure 2a–c shows the evolution of the fundamental mode of the hybrid SOI-Sb_2_Se_3_ waveguide as the thickness is increased. Maps show the simulated field profiles, with the number in the top left indicating the mode overlap with the original SOI waveguide mode without any Sb_2_Se_3_ and the percentage in the top right indicating the percentage of the mode intensity contained in the Sb_2_Se_3_ layer. Corresponding plots of the mode overlap and the percentage of energy localized inside the PCM slab are shown in Figure 2c,d against the Sb_2_Se_3_ thickness. For Sb_2_Se_3_ in the amorphous state, where the refractive index is lower than that of silicon, we observe that the mode remains very closely matched to the SOI waveguide with mode overlap better than 0.95. For the crystalline Sb_2_Se_3_, the mode is coupled more into the higher-refractive-index PCM and therefore starts to deviate more significantly from the SOI mode. We see a reduced mode overlap of 0.86 with the SOI mode for 100 nm crystalline Sb_2_Se_3_. The coupling of mode intensity to the PCM layer increases from around 2% for the 20 nm thin layer in both states up to, respectively, 11.4% and 34.5% for the 100 nm Sb_2_Se_3_ in amorphous and crystalline states.

### Characterization of SWG Losses

In order to evaluate the waveguide characteristics with increasing Sb_2_Se_3_ thickness and for amorphous/crystalline states, we first performed a series of experimental waveguide loss studies. The fabricated chips feature a series of SWG devices functionalized with slabs of Sb_2_Se_3_ PCM ranging from 0 to 800 μm in length. Insertion loss measurements were performed at 1550 nm wavelength to determine the waveguide loss versus length, for both as-deposited amorphous samples and hot-plate-crystallized Sb_2_Se_3_ (see Methods). Results are presented in Figure 2d,e for the crystalline and amorphous Sb_2_Se_3_, respectively. All results for PCM cladded devices were normalized to a straight SOI rib waveguide of the same total length, represented by the data point at zero length of the Sb_2_Se_3_ slab. Error bars were obtained from the measured variation over three different waveguides on the same chip for each length of the Sb_2_Se_3_ patch.

Figure 2d shows that losses increase exponentially with the length of Sb_2_Se_3_, following the Beer–Lambert law, which on a dB scale shows a linear trend with the slope equal to the loss coefficient α in dB/μm. In the amorphous state, losses stay very low for increasing thickness and length of Sb_2_Se_3_ and, in fact, the waveguide loss is reduced compared to the reference waveguide without PCM, resulting in a negative loss coefficient α. The reduced loss can be attributed to the increase in the waveguide cross section, resulting in reduced surface scattering compared to the original SOI device.

Waveguide loss coefficients α were extracted using linear fits to the experimental data, as shown by the lines in Figure 2d,e, and the resulting values are presented in Figure 2f against Sb_2_Se_3_ thickness, for both the crystalline (red dots) and amorphous (blue diamonds) states. Corresponding values for α in the crystalline state are tabulated in Table 1 and plotted in Figure 2f. The loss coefficient in the crystalline state shows a power-law dependence on the Sb_2_Se_3_ thickness with an exponent of 2.8. In general, waveguide losses consist of surface and volume contributions. The Payne–Lacey model is often used for homogeneous waveguides and assumes sidewall roughness as the predominant cause of scattering. Within this model, the scattered intensity scales quadratically with the surface roughness parameter.^31^ The Rayleigh–Gans volume scattering by small polycrystalline domains gives a scattering cross section that scales quadratically with the scatterer volume.^32^ Coupling of the waveguide mode with the high-index crystalline Sb_2_Se_3_ further increases the loss coefficient as more mode intensity is increasingly concentrated inside the PCM layer for a larger thickness.

**Table 1:** Experimental Propagation Loss α, Optical Phase Shift Δϕ, and Calculated FOM for SOI Waveguides with Different Thicknesses of Embedded Sb_2_Se_3_

Sb_2_Se_3_ thickness (nm)	α (dB/ μm)	Δϕ (rad/ μm)	FOM (rad/dB)	*L*_π_ (μm)
20	0.003 ± 0.001	0.116 ± 0.002	39 ± 12	27.1
40	0.019 ± 0.002	0.223 ± 0.002	11.7 ± 1	14.1
60	0.057 ± 0.004	0.306 ± 0.004	5.4 ± 0.4	10.3
80	0.120 ± 0.020	0.600 ± 0.002	5.0 ± 0.7	5.2
100	0.280 ± 0.060	0.563 ± 0.007	2.0 ± 0.4	5.6

### Characterization of Switching-Induced Optical Phase Shift in Silicon-Sb_2_Se_3_ MZIs

**Figure 3:**
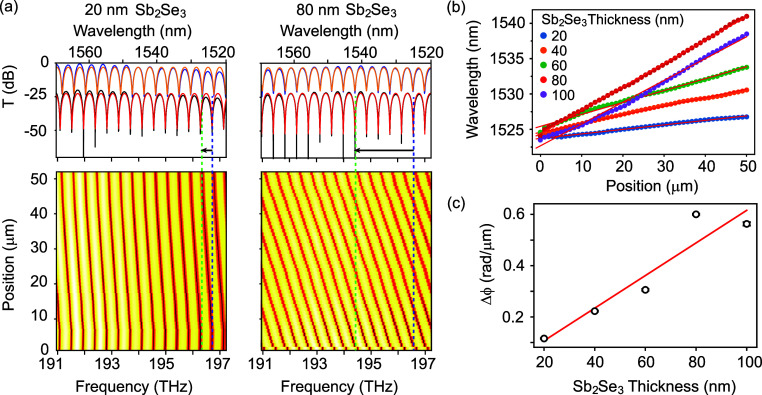
(a) Spectra of MZIs before and after crystallization (top panels) and maps taken after every 1 μm of crystallization (bottom panels) by direct laser writing over 50 μm of Sb_2_Se_3_, for devices with Sb_2_Se_3_ thicknesses of 20 and 80 nm. Black/blue curves in the top panel: experimental spectra; red/orange curves: model fits. Vertical dashed lines indicate shift of the selected mode with initial wavelength around 1524 nm. (b) Wavelength position extracted from fits to experimental spectra for MZI devices with Sb_2_Se_3_ thickness between 20 and 100 nm. Red lines: linear fits to data. (c) Extracted values of slope Δϕ plotted against the Sb_2_Se_3_ thickness. Red line: linear fit.

The induced optical phase shift was characterized by using the spectral response of asymmetric MZI devices containing a patch of Sb_2_Se_3_ in one of the arms. Measurements were taken of spectra between 1520 and 1570 nm, while Sb_2_Se_3_ was incrementally crystallized using direct laser writing in 1 μm steps, using the setup described in the Methods section. Results are shown in Figure 3a for MZI devices with the 20 and 80 nm thick Sb_2_Se_3_ layers; results for the other layers are presented in the Supporting Information Figure S7. The top panels show the experimental transmission spectra of the device before (black) and after (blue) switching.

Fits using the typical periodic MZI comb function are also presented by the red and orange curves, respectively, allowing one to extract precise values for the free spectra range, wavelength shift, and contrast, taking into account the contribution from all peaks in the spectrum. The fitting procedure is discussed in more detail in the Supporting Information Figure S6.

Full experimental maps of the spectra versus switching length along the Sb_2_Se_3_ slab are shown in the bottom panels in Figure 3a, showing the results of 51 individual spectra measured as the direct-write laser was scanned along 50 μm of the PCM patch.

For each device, we selected a transmission minimum around 1525 nm (196.6 THz) and followed the position of this peak along the switching position using the fitted spectra to provide the most accurate results. The wavelength shift versus position is shown in Figure 3b for all five thickness values of the Sb_2_Se_3_ layer. For all samples, we see a wavelength shift of this feature proportional to the length of the switched region, which could be fitted (lines) to obtain a wavelength shift of Δλ per unit of length. The wavelength shift Δλ is converted to a phase shift Δϕ by considering the fitted free spectral range (FSR) of the MZI spectra, which is equivalent to 2π radians of phase shift
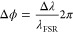
1

Figure 3c presents the resulting values of Δϕ for the set of devices with different Sb_2_Se_3_ layer thicknesses. Similar results were obtained for two other sets of devices, containing a 10 nm thin SiO_2_ buffer layer between the silicon waveguide and the PCM, as presented in the Supporting Information Figures S8 and S9. Altogether, these results support a trend showing a linear increase of the induced optical phase shift Δϕ with Sb_2_Se_3_ thickness in the range from 20 to 100 nm. In terms of the FSR itself, fitting of the frequency transfer function of the MZI, presented in the Supporting Information Figure S10, shows an ∼1.2% reduction of the FSR frequency after switching of a 50 μm long slab of the 100 nm thick Sb_2_Se_3_ layer. The mode at 1525 nm wavelength corresponds to the 475th FSR in the frequency spectrum, with the FSR being 0.41385 Thz, or 3.208 nm at 1525 nm wavelength.

### Device FOM

To quantify the overall performance taking into account both the optical phase shift and losses, we use a device FOM introduced previously in ref (16), defined by the ratio of the optical phase shift over the device loss
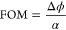
2Compared to the conventional materials FOM Δ*n*/Δ*k*, which only takes into account bulk properties, the device FOM provides a more useful value of the performance in a waveguide geometry. Given the low intrinsic values for the material losses in antimony-based PCMs,^16^ the materials' FOM diverges in the near-infrared,^1^ requiring the use of a more realistic performance figure such as defined in eq 2. The device FOM allows us to consider not just the change in refractive index between the two phases of the materials but also to take into account the inherent losses of the material and structure to gain a full appreciation of how useful this material is in this configuration.

For the FOM, we use the propagation loss of the crystalline phase of Sb_2_Se_3_, as this is typically much higher than in the amorphous phase and is the performance limiting value. Performing this calculation for our range of thicknesses, we arrive at the results presented in Table 1. The device FOM reaches a value around 39 for the thinnest Sb_2_Se_3_ layers under study, consistent with earlier reported findings.^16^ Increasing the thickness of the PCM results in a sharp drop of the FOM because of the additional waveguide losses, which increase much more rapidly with thickness than the induced phase shift. However, as long as losses of the order of 1 dB can be tolerated, there is much to gain in terms of device length by increasing the PCM thickness. The reduction in device length can be appreciated from the calculated values for *L*_π_ in Table 1. A 5-fold reduction of *L*_π_ from 27.1 μm down to 5.2 μm is obtained by increasing the Sb_2_Se_3_ thickness from 20 to 80 nm at the cost of 0.62 dB insertion loss over the device length compared to 0.08 dB for the 20 nm thin film. Table 1 thus demonstrates the trade-off between the benefit of increasing the thickness of Sb_2_Se_3_, increased modulation, and the disadvantage of increased propagation losses.

### Digital Patterning of MMIs Using Direct Laser Writing

Having established the effect of increased Sb_2_Se_3_ layer thickness on the induced optical phase shift in an MZI configuration, we proceed with our investigation of the digital pixel patterning of MMIs for programmable multiport photonic circuits. Experiments were done starting from simulated pixel patterns, which were obtained using a forward iterative optimization similar to that used in refs (26, 27). Experimentally, transmission of both output ports was measured simultaneously using a custom-built dual-fiber probe.^17^ Intensity at both outputs was measured during the direct laser writing and was recorded for each pixel, allowing extraction of total device throughput and transmission contrast between the output ports. In our studies, we considered both amorphous pixels on a precrystallized Sb_2_Se_3_ device, similar to a previous work,^17^ and crystalline pixels on an as-grown amorphous Sb_2_Se_3_ layer.

#### Amorphous Pixels on Crystalline MMI

**Figure 4:**
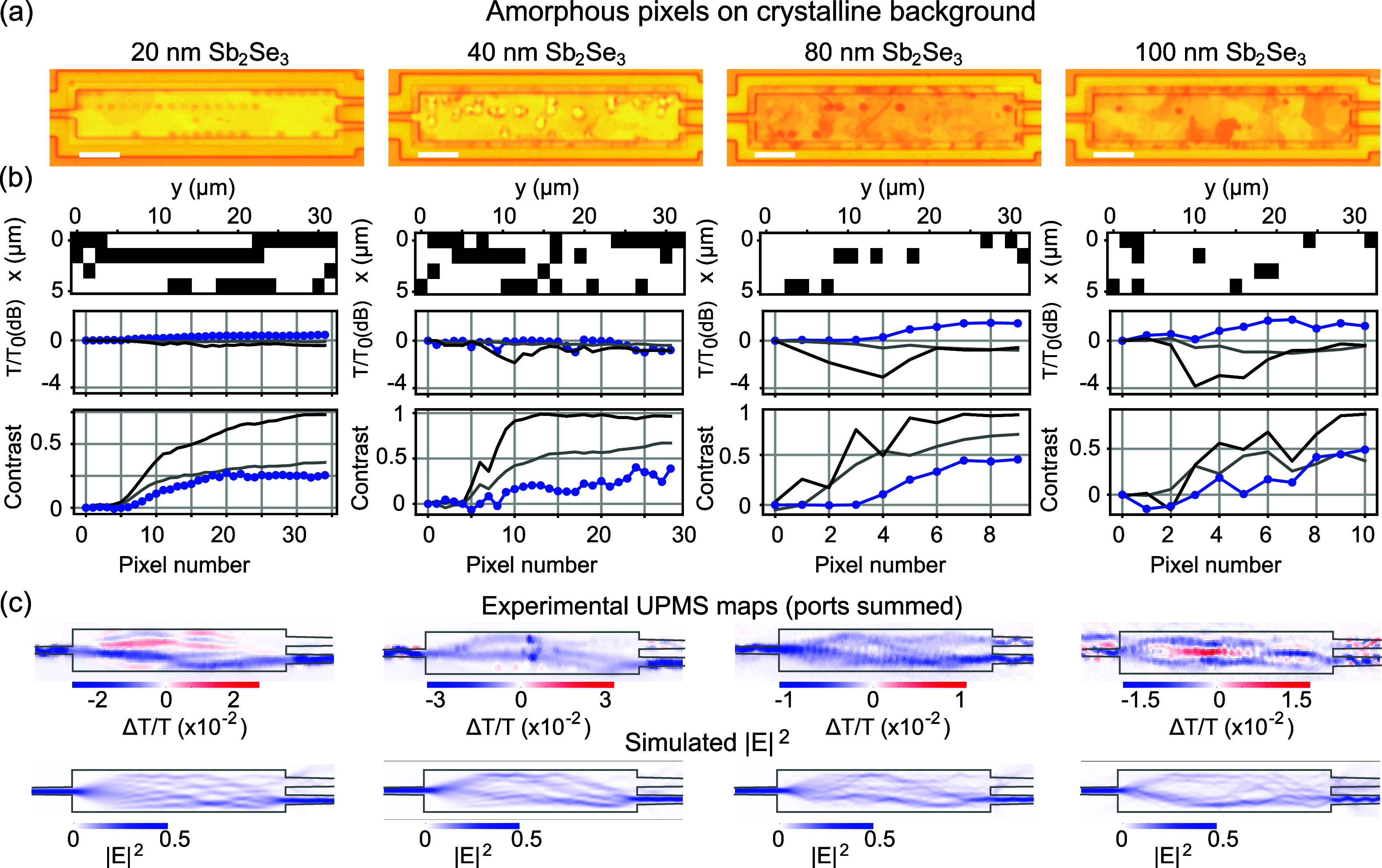
(a) Optical microscopy images of patterned MMIs for Sb_2_Se_3_ samples of 20, 40, 80, and 100 nm thickness for amorphous pixels written on a precrystallized PCM using direct laser writing. Scale bar is 5 μm. (b) Designed perturbation maps showing switched pixels (top). Total transmission over both output ports *T* = *T*_btm_ + *T*_top_, normalized to transmission before switching, *T*_0_ (middle), and contrast between top and bottom output ports (*T*_btm_ – *T*_top_)/*T* (bottom), shown versus the switched pixel number. Experimental data (blue dots) and simulations with 1.5 and 0.75 μm pixel size (dark-/light-gray lines). (c) UPMS maps of total port transmission, Δ*T*/*T* (top), and calculated near-field intensity maps |*E*|^2^ over the MMI devices.

Figure 4 shows the results for four selected MMIs with the Sb_2_Se_3_ thickness ranging between 20 and 100 nm. The samples were precrystallized using a hot plate, and amorphous pixel patterns were programmed by scanning a direct-write laser over the area. Optical microscopy images are presented in Figure 4a, and the target design patterns are shown in (b). Figure 4b also shows the overall device throughput *T* summed over both bottom (*T*_btm_) and top (*T*_top_) output ports, normalized to the initial value before patterning *T*_0_, as well as the port contrast, defined as (*T*_btm_ – *T*_top_)/*T*_0_.

Application of the pattern results in an increased transmission of the bottom output. A lower contrast is seen for the experiments compared with the simulations. We attribute the different contrast to the smaller pixel size in the experiment of Figure 4a compared to the design of (b), which resulted in weaker perturbation strength in the experiments than in the model. To take this effect into account, we recalculated the device response taking into account a pixel size of 750 nm, as shown by the gray lines in Figure 4b. Additionally, Supporting Information Figure S13 considers differences due to partial etching of the devices by Ar-ion plasma treatment. Another factor of importance is the switching depth that can be achieved. In our earlier studies, switching depths exceeding 200 nm were achieved for direct-write amorphization pulses in crystalline Sb_2_Se_3_ thin films^24^; therefore, we expect the devices under study to be in the regime where the entire thickness of the PCM is switched.

Overall, taking into account any differences in experimental and simulated response, we observe that many fewer pixels are needed to induce the same change for the thicker Sb_2_Se_3_ layers. For the device throughput, we see that simulations show the opposite effect than experiments, where an improvement of transmission is obtained by pixelating. This can be explained by the fact that, in our simulations, we do not take into account the scattering-induced losses in the crystalline state. The simulation therefore overestimates the throughput of the unperturbed devices.

#### Ultrafast Photomodulation Spectroscopy (UPMS)

Whereas the port transmissions provide important information about the effect of digital patterning of the device, additional information can be gained by looking at the local field distribution inside the device. In this work, we have investigated for the first time the flow of light inside MMIs with PCMs using UPMS, a technique capable of mapping the flow of light in space and time.^33^ In UPMS, an ultrafast pump laser focus is scanned across the device, while the transmission through the waveguide is monitored using a pulsed probe laser.^34^ Previously, we obtained results for waveguides patterned by etching of holes into the silicon.^27^Figure 4c shows the experimental UPMS maps taken of the patterned devices, where the differential transmission Δ*T*/*T* is shown summed over both output ports. A detailed model explaining the UPMS response can be found in (34). In short, a negative Δ*T*/*T* response indicates a reduced port transmission, while a positive value means an increased port transmission in the presence of the ultrafast pump laser.

For a device with high throughput, the summed-port response is predicted to follow closely the local near-field intensity profile in the device.^34^ We compare our results directly to the calculated near-field intensity |*E*|^2^, presented in Figure 4c, bottom panels. A Gaussian smoothing filter was applied to take into account the experimental resolution, blurring out some of the finer features but retaining the light flow profiles in the different patterned MMIs. The maps show that different pixel patterns result in varying flow profiles, as is the case for the experimental UPMS maps. In the absence of losses, the port-summed UPMS maps should ideally show only a perturbation-induced loss and hence an overall blue color. For the 40 and 80 nm Sb_2_Se_3_ layers, this is indeed observed, and the demonstrated flow patterns are in good overall agreement between the experimental and simulated maps. Branched flows are typically observed in weakly perturbed waveguides^35^ and are indicative of the regime of weak forward scattering.

The presence of positive Δ*T*/*T* response in the maps taken for the 20 and 100 nm Sb_2_Se_3_ MMIs in Figure 4c is indicative of positions on these MMIs where an additional perturbation increases the throughput. This indicates that the device has additional loss channels related to a mismatch between the designed and experimentally applied pixel patterns. For example, for the 100 nm Sb_2_Se_3_ device, the calculated near-field map shows that the light splits into two branched flows at the top and bottom of the device. This branching into a top and bottom flow is also seen experimentally. Additionally, we see that placing the experimental perturbation in the middle of the MMI improves the throughput, indicating that some of the light did not follow the designed flow pattern, but the extra pump-induced perturbation acts to reroute some of the misdirected light back to the outputs. Similarly, for the 20 nm Sb_2_Se_3_, it appears that the long row of pixels at the top of the device was insufficient in rerouting the light; placing the perturbation in this area also improves the routing of light toward the MMI outputs.

#### Crystalline Pixels on As-Grown Amorphous MMI

The method of direct laser writing of amorphous pixels on a crystalline background, such as that shown in Figure 4, results in small pixels with well-defined edges. This can be understood as the time scales associated with vitrification are of the order of tens of nanoseconds, much shorter than the times for lateral heat diffusion. However, this approach requires the entire PCM layer to be crystallized, which means that most of the PCM is in the state where losses are increased compared to the original device.

**Figure 5:**
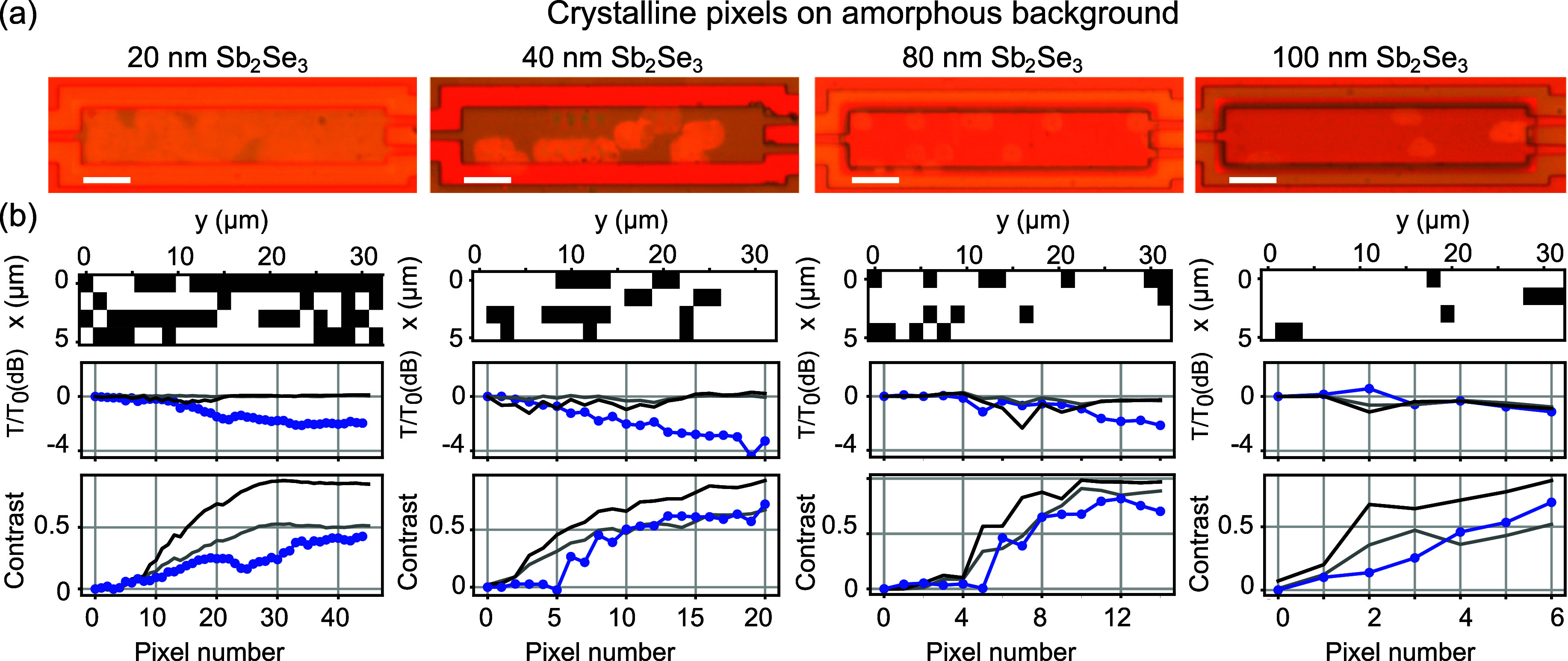
(a) Optical microscopy images of patterned MMIs for Sb_2_Se_3_ samples of 20, 40, 80, and 100 nm thickness for crystalline pixels written on as-grown amorphous PCM using direct laser writing. Scale bar is 5 μm. (b) Designed perturbation maps showing switched pixels (top). Total transmission over both output ports *T* = *T*_btm_ + *T*_top_, normalized to transmission before switching, *T*_0_ (middle), and contrast between top and bottom output ports (*T*_btm_ – *T*_top_)/*T* (bottom), shown versus the switched pixel number. Experimental data (blue dots) and simulations with 1.5 and 1.0 μm pixel size (dark-/light-gray lines).

Next, we investigate the possibility of starting from the as-grown, amorphous device and inducing patterning by the selective crystallization of pixels. Crystallization offers limited spatial control because of lateral heat diffusion and crystallization dynamics, generally resulting in larger pixel size compared with amorphous pixels resulting from rapid vitrification. Here, we explore this route experimentally as shown in Figure 5. Patterns were designed numerically before the experiment, following the same methodology as before but using crystalline pixels on an amorphous PCM background. Direct laser writing in this configuration results in much larger crystallized regions of around 1 μm, as can be seen in Figure 5a for all of the Sb_2_Se_3_ layer thickness values, which is expected from the different time scale allowing for significant lateral heat diffusion and crystal growth. In the 20 nm Sb_2_Se_3_ layer, where patterns exceeding 40 pixels were required, the individual laser-written spots are seen to fuse into a semicontinuous crystalline region with very little remaining structure. For the other three devices, distinct pixels can still be identified.

The throughput and switching contrast of the devices are shown in Figure 5b. The devices show a good switching contrast owing to the larger pixel size than in the amorphous case. However, the large pixel size limits the number of pixels that can be written for the 20 nm Sb_2_Se_3_ device, where the contrast is reduced due to the loss of pattern structure due to pixel fusion. As can be seen in the *T*/*T*_0_ trends, the writing of crystalline pixels on an amorphous PCM background lowers the total transmission by up to 4 dB compared to the original device; this is in agreement with the expectation based on additional losses in the crystalline state. Simulations underestimate this device loss, again because of the absence of scattering by polycrystalline domains. The 80 and 100 nm Sb_2_Se_3_ devices in this set of devices show the best agreement with simulations, as the pixel size is very well matched and the number of pixels is sufficiently small to allow for each pixel to act effectively on its own. UPMS measurements were attempted on this set of devices using the same configuration as in Figure 4c, but the effectiveness of the technique was strongly reduced by a very low photomodulation response for Sb_2_Se_3_ in its amorphous state. Further work is needed to more systematically explore this difference in response, which goes beyond the scope of the current paper.

### Discussion

**Figure 6:**
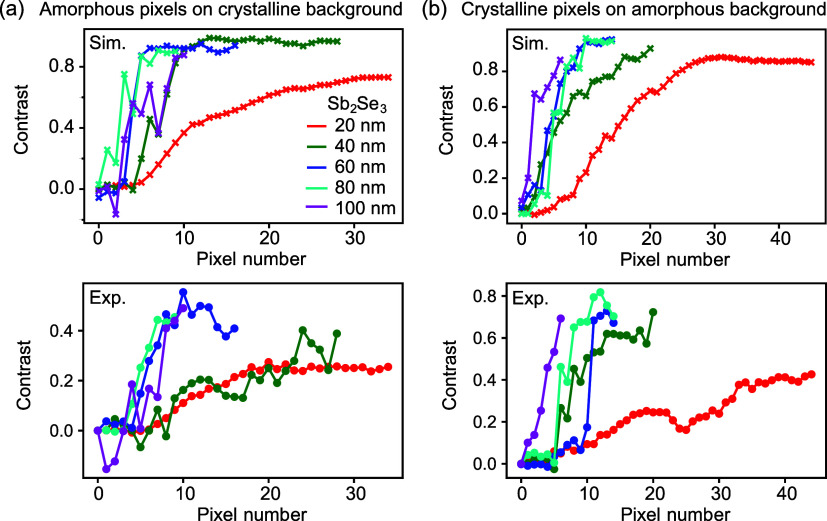
Contrast between bottom and top output ports against pixel number for five patterned MMIs with different Sb_2_Se_3_ thicknesses between 20 and 100 nm, for amorphous pixels on a crystalline background (a) and crystalline pixels on an as-grown amorphous background (b). Top graphs, simulation results for design patterns; bottom graphs, experimental results.

Combining our results for the amorphous and crystalline pixel patterns, Figure 6 summarizes the port contrast against the pixel number for the investigated devices. This set includes the 60 nm Sb_2_Se_3_ layer device, whose data are included in the Supporting Information Figure S13. The results support the overall conclusion that thicker layers of the low-loss PCM Sb_2_Se_3_ allow for increased perturbation strength, reducing the number of pixels needed to achieve a given splitting ratio using direct-write digital patterning of an MMI device. Results for the amorphous pixels were somewhat lower mainly because of the smaller pixel size, whereas crystalline pixels provided an inherently much larger pixel size, which posed challenges for defining intricate designs with many pixels; however, for the thicker Sb_2_Se_3_ layers, it was otherwise very effective in routing light using only a small number of pixels. The results for the digital patterning of MMIs are in agreement with the larger induced phase shift observed in our MZI calibration, which formed the basis of our numerical pattern designs.

Our work has been done using research-grade instruments that have not been optimized for speed. Fast automated programming will be possible using industrialized tools, which could enable chip-level or even wafer-scale direct laser writing of photonic devices. Some impressive steps in this direction have been shown recently using a high-throughput commercial direct-write system.^18,28^ Reduction of the number of required pixels, as well as the use of relatively large pixel sizes to achieve the desired effects, will contribute to improving the write times. We believe that this is an important advantage of our digital patterning approach as opposed to topological inverse design with extremely high-resolution features that require much longer write times. Partial crystallization^25^ or interface switching^36^ may also be used for significantly enhanced switching speed. The surrounding materials’ thermal design can also substantially improve switching speeds by, for example, reducing the threshold energy for switching.^37^

In the range of potential applications, we see particular promise in offline programming for diversification of chips coming from a high-volume manufacturing process.^14^ Another real-world example could be router or ROADM technologies; these normally have a slow path where a connection is preserved for longer times. A range of emerging technologies such as programmable neural networks or quantum simulators rely on programmable elements for weight banks or operators.^2,38^ Such types of reprogrammable devices do not necessarily require very fast switching, and an endurance of thousands up to millions of cycles may already be sufficient for real-world applications.^15,24,25,39^

## Conclusions

In conclusion, we have investigated the dependence of the optical switching efficiency of layers of different thicknesses of the low-loss PCM Sb_2_Se_3_ when integrated onto a standard 220 nm silicon photonics platform. Propagation loss measurements on SWGs were combined with precise measurements of the induced optical phase shift in an MZI configuration to arrive at a device FOM for each layer thickness. A large, 5-fold increase of the induced phase shift was observed by increasing the PCM thickness from 20 to 80 nm, resulting in a device length reduction *L*_π_ from 27.1 μm down to 5.2 μm. This strong reduction opens possibilities for new types of ultracompact photonic devices. The reduced footprint has to be traded off against increased optical losses, increasing to an insertion loss of around 0.62 dB for the 80 nm thick Sb_2_Se_3_ layer. The calibration of the optical phase shift was subsequently used to design new types of programmable photonic routers based on direct-write digital patterning of an MMI. The application of pixel patterns was shown to result in branched flows of light streaming from the input to the selected output, which were experimentally visualized using UPMS mapping.

The increased thickness of Sb_2_Se_3_ resulted in a reduction of the required pixels from larger than 30 to less than 10 per patterned device, which significantly reduced the complexity of the scheme. Amorphous pixels on a crystalline background were shown to offer precise resolution and control over the pixels, however at the cost of increased losses in the precrystallized background. In comparison, crystalline pixels on an amorphous background were demonstrated as an alternative, showing larger pixel sizes with reduced control but stronger perturbations per pixel, which worked particularly well for thicker Sb_2_Se_3_ layers. The demonstrated new capability has relevance in the postfabrication tuning of silicon photonic devices and may hold promise for achieving reconfigurable and free-form programmable devices for optical processor technology, photonic AI hardware, and quantum computing.

## Methods

### Waveguide Fabrication

SOI waveguide devices were fabricated by deep-UV lithography on 200 mm diameter SOI wafers using the Cornerstone silicon photonics foundry at the University of Southampton, UK. In a first exposure, photonic rib waveguides of 120 nm height were fabricated onto the 220 nm SOI platform by using an etching step. A second deep-UV exposure was used to define the open windows in a photoresist at selected locations on the wafer to allow subsequent etching and PCM deposition. The wafer was cleaved into chips.

Individual chips were further processed by loading them into a sputter tool (AJA Orion). A first Ar-ion etch was done using 30 W of RF bias in argon in order to remove any residual resist. Subsequently, the Sb_2_Se_3_ and cladding stack were deposited without breaking the vacuum. For results presented in the main article, Sb_2_Se_3_ was deposited directly onto the silicon waveguide. A second set of samples was produced including a 20 nm thin SiO_2_ buffer layer between the silicon waveguide and Sb_2_Se_3_. The Sb_2_Se_3_ layer was sputtered from a stoichiometric Sb_2_Se_3_ target (Testbourne) with a thickness proportional to the sputtering time. A 20 nm thin SiO_2_:ZnS (80:20 ratio) dielectric was deposited as a thin protective layer. The photoresist was then removed using NMP, acetone, and IPA. The final step was to clad the sample, which was performed by sputtering 100 nm of SiO_2_:ZnS over the entire chip.

### Crystallization Using Thermal Annealing

Thermal annealing was performed for selected devices using a hot plate. To crystallize the Sb_2_Se_3_ integrated into photonic chips, they were heated to 190 °C for 10 min, after which they were inspected using an optical microscope.

### Optical Spectroscopy

Insertion loss spectra of as-grown amorphous and precrystallized samples were done using a standard insertion loss measurement system. Studies involving direct laser writing with sequential spectroscopy were done using a customized setup including a direct-write diode laser (Vortran Stradus) at a 638 nm wavelength and 160 mW peak power. Short digital trigger pulses were generated by using a Berkeley Nucleonics pulse generator in order to produce a variety of pulse lengths and powers. A 50×, 0.5 numerical aperture objective was used (Mitutoyo), and the objective position was scanned using a closed-loop piezo nanopositioning system.

A broadband swept tunable laser source (Keysight N7778C) was used for sequential scans of MZIs in combination with a multiport power meter (Keysight N7744A). For the two-port output detection, a custom-built two-fiber arm was used to simultaneously collect both outputs. The setup was controlled by using a computer interface (Labview).

### UPMS

For ultrafast mapping, we use a setup similar to that reported in refs (33) and (34). An ultrafast fiber laser (Menlo) at 1550 nm wavelength was used as the probe at 100 MHz repetition rate. Part of its output was quadrupled to 390 nm using two second harmonic crystals, resulting in several mW optical power at the UV wavelength. The UV output was amplitude-modulated at 10 MHz using an acousto-optic crystal and was focused on the top of the device under test using a 0.5 NA objective (Mitutoyo) with a closed-loop nanopositioning system for scanning (Smaract). Light was detected using an APD (Thorlabs) and a lock-in amplifier (Zurich Instruments).

## Data Availability

Supporting data used in this work is openly available from the University of Southampton repository at doi.org/10.5258/SOTON/D3353.

## Supporting Information Available

Supporting simulation results, experimental procedures, description of model fits, and additional experimental data (PDF)
