# Supplementary material for: Ultracompact Programmable Silicon Photonics Using Layers of Low-Loss Phase-Change Material Sb2Se3 of Increasing Thickness
Source: ACS Photonics. 2025 Mar 7;12(3):1382–91. doi: 10.1021/acsphotonics.4c01789 (PMC11926964; doi:10.1021/acsphotonics.4c01789)
Supplement: Supplementary file 1 — ph4c01789_si_001.pdf [file ph4c01789_si_001.pdf]

Supporting Information: Ultracompact programmable silicon photonics using layers of low-loss phase-change material Sb_2Se_3 of increasing thickness.

Sophie Blundell,[†,‡] Thomas W. Radford,[‡] Idris A. Ajia,[‡] Daniel Lawson,[‡] Xingzhao Yan,[†] Mehdi Banakar,[†] David J. Thomson,[†] Ioannis Zeimpekis,[†,¶] and Otto L. Muskens*,[‡]

†*Optoelectronics Research Centre, University of Southampton, Southampton, SO17 1BJ, United Kingdom*

‡*School of Physics and Astronomy, University of Southampton, Southampton, SO17 1BJ, United Kingdom*

¶*Electronics and Computer Science, University of Southampton, Southampton, SO17 1BJ, United Kingdom*

E-mail: o.muskens@soton.ac.uk

Phone: +44 (0)23 80593911

1 Simulation

Computational modelling of PICs is a useful tool for designing PICs as well as confirming experimental results. Modelling the propagation of beams through waveguiding structures is a non-trivial problem due to the complexity of calculating propagating modes and due to

the need to impose boundary conditions upon time-dependent Maxwell's equations. This could be addressed using the finite-difference beam propagation method (BPM) as set out by Xu and Huang in 1995,[1] but this BPM approach relies on a slowly-varying envelope approximation which may not be appropriate for discontinuous media or high-index contrast materials, both of which apply to including a PCM in a waveguide structure. Instead, a finite-difference time-domain (FDTD) approach can be taken, as first introduced by Yee in 1966.[2] The time-domain aspect of FDTD allows multiple frequency analysis, enabling a broadband response to be calculated, as well as providing more intuitively understandable simulations. An extra benefit of using FDTD is that the mesh structure used over the physical structure does not have to be uniform, so can more realistically model a PIC. FDTD involves discretising the overall structure into "cells", and solving Maxwell's equations for each cell in turn in the direction of propagation. The main downside of using an FDTD approach is that the size of device that can be modelled is limited by the computational resources available. Such simulation can be made more efficient, and more practically possible for larger devices, by using a 2D simulation as opposed to a full 3D simulation. Where light is propagating solely in-plane within a PIC and assuming that there is minimal coupling between modes, the effective refractive index method can be employed, which uses the effective index of the first cell, calculated by collapsing the 3D geometry, to calculate the effective index for the next cell, again in the direction of propagation.[3] Using this method is a way to simulate a 3D structure by considering only 2D propagation which essentially reduces the dimensionality of the problem and is sometimes called 2.5D variational FDTD as it offers comparable accuracy to a 3D simulation with a similar run time to a 2D simulation.

The Lumerical MODE variational FDTD software package was used to simulate the silicon photonic waveguides in this work. The structure shown in Fig. S1 was constructed, featuring an Sb_2Se_3 block. This waveguide cross-section has a 3 μm thick SiO_2 substrate, 100 nm thick silicon layer and 220 nm thick silicon rib waveguide. For the purpose of simulating a waveguide with this cross-sectional geometry, in three dimensions the model waveguide has

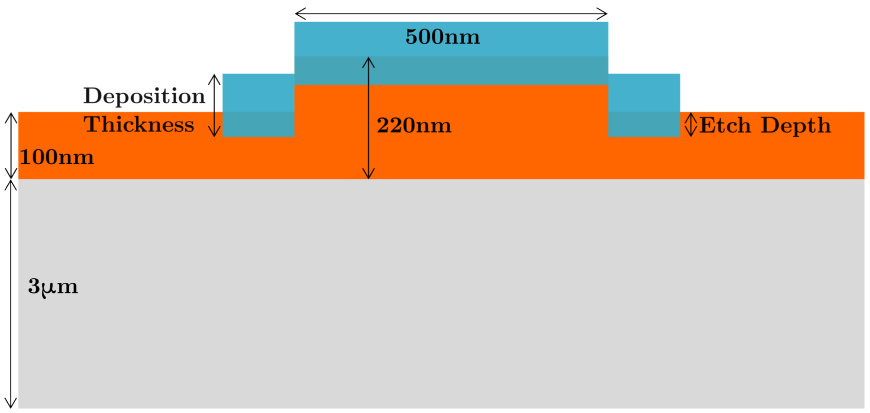

Figure S1: The structure simulated for a variety of deposition thicknesses and etch depths of Sb_2Se_3 , featuring a silicon rib waveguide and silica substrate.

a length of 200 μm, with the Sb_2Se_3 block spanning the centre 50 μm of this. An X-normal eigenmode solver is added to the simulation, centred on the rib waveguide and Sb_2Se_3 block, with a size of $2 \times 1 \;\; \mu m^2$ in the y and z dimensions respectively. With Sb_2Se_3 layered on top of the waveguide thickness values of Sb_2Se_3 between 20 nm and 100 nm were simulated. A source of light, injected in the positive x-direction at 10 μm from the end of the waveguide and with a wavelength of 1550 nm allows measurement of the change in transmission of this light when the Sb_2Se_3 block is altered.

As a measure of the difference in refractive index profile of the structure, $n_{\text{eff}}$of fundamental mode of the light injected is plotted for Sb_2Se_3 in both its crystalline and amorphous phases in Fig. S2 for the six values of Sb_2Se_3 layer thickness on the surface of the waveguide. It can be seen that for thicker layers the difference in n_{eff}increases, meaning that a larger change in transmission for switching the material's state can be achieved with a thicker layer. While n_{eff}increases with thickness of Sb_2Se_3 , there is a limit to this trend beyond a thickness of 60 nm. This limit is attributed to the way that the light is interacting with the Sb_2Se_3 , via evanescent coupling, which normally sees amplitude of light rapidly decay with distance.

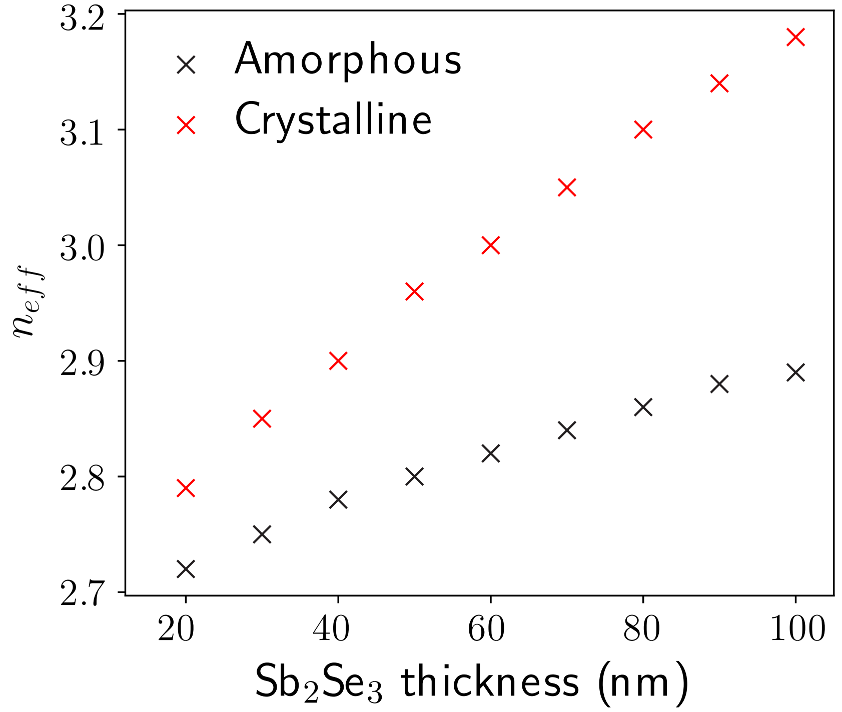

Figure S2: Effective refractive index of fundamental modes for different thicknesses of Sb_2Se_3 deposited on the surface of a silicon waveguide in the crystalline and amorphous states.

1.1 Etched Waveguides for Enhanced Modulation

Before the PCM deposition process it is possible to etch into the surface of a silicon waveguide. In doing so, prior to deposition of Sb_2Se_3 , the PCM can effectively replace part of the silicon waveguide, making the mode overlap larger and thus the change in transmission enacted by switching the Sb_2Se_3 larger. This effect can be simulated using Lumerical FDTD by changing the structure of the waveguide model. In this case, an eigenmode solver is used, centred again on the waveguide and Sb_2Se_3 block, and the $n_{\text{eff}}$of the fundamental mode recorded for etch depths and deposition thicknesses between 0 and 100 nm. Investigating the parameter space for etch depth and deposition thickness shows, in Fig. S3, that the highest change in effective refractive index occurs for the 100 nm deposition with an etch depth of 100 nm. The dashed line in this Figure indicates the design conditions used for the MMI optimization. Experimentally, the etch depth does not reach this condition, as indicated by the SEM cross-sections. The other limiting case of zero etching is indicated by

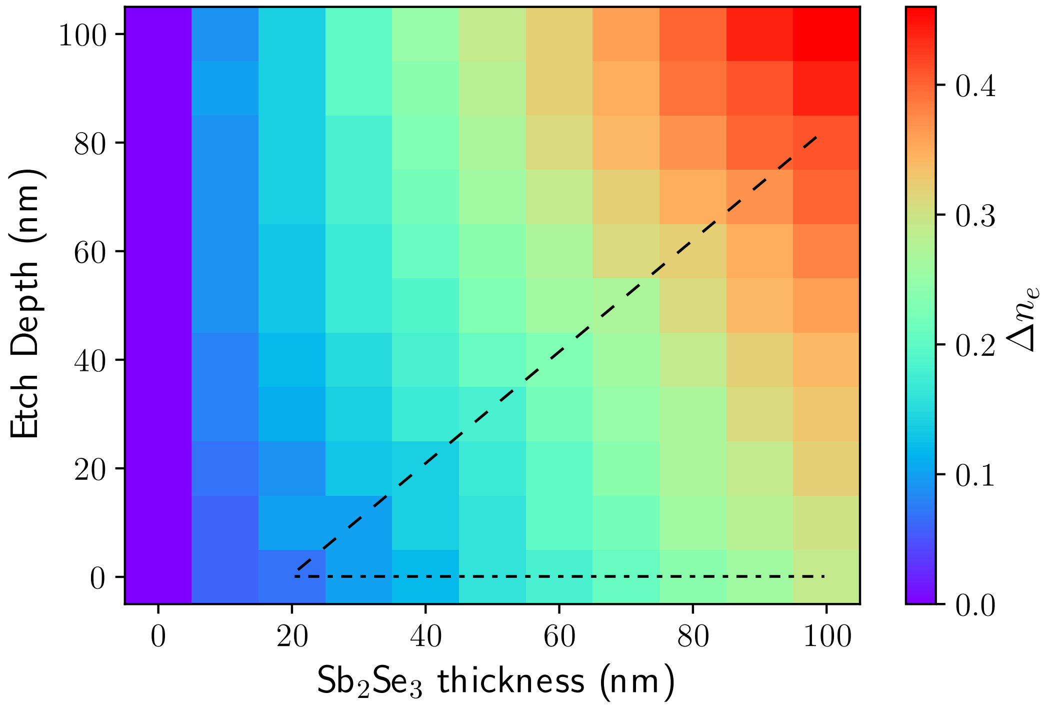

Figure S3: Change in effective refractive index between the crystalline and amorphous states of a waveguide embedded with Sb_2Se_3 when the material thickness and depth of the etch performed prior to Sb_2Se_3 deposition are varied. Dashed line: design used for MMI optimization; dash dotted line: no etch.

the dash-dotted line in Fig. S3. The corresponding values for these two limiting cases are plotted in Fig. S4, corresponding to the cross sections of the map of Fig. S3. We see a similar trend in Δn_{eff} against thickness of Sb_2Se_3 for etched and unetched waveguides, indicating a that etch depth mainly affects the absolute value of the switching. Quantitatively, the difference in Δn_{eff} between the fully etched and zero etched conditions is equivalent to an variation in the Sb_2Se_3 layer thickness of up to 20 nm for the thickest layers in this study.

1.2 Silicon Chip Fabrication

The chips used in this project were fabricated using Deep-UV projection lithography, where a UV beam is scanned directly over the photolithography mask, projecting the chip design onto a wafer. Deep-UV lithography is typically used in industry due to its rapidity, scalability and

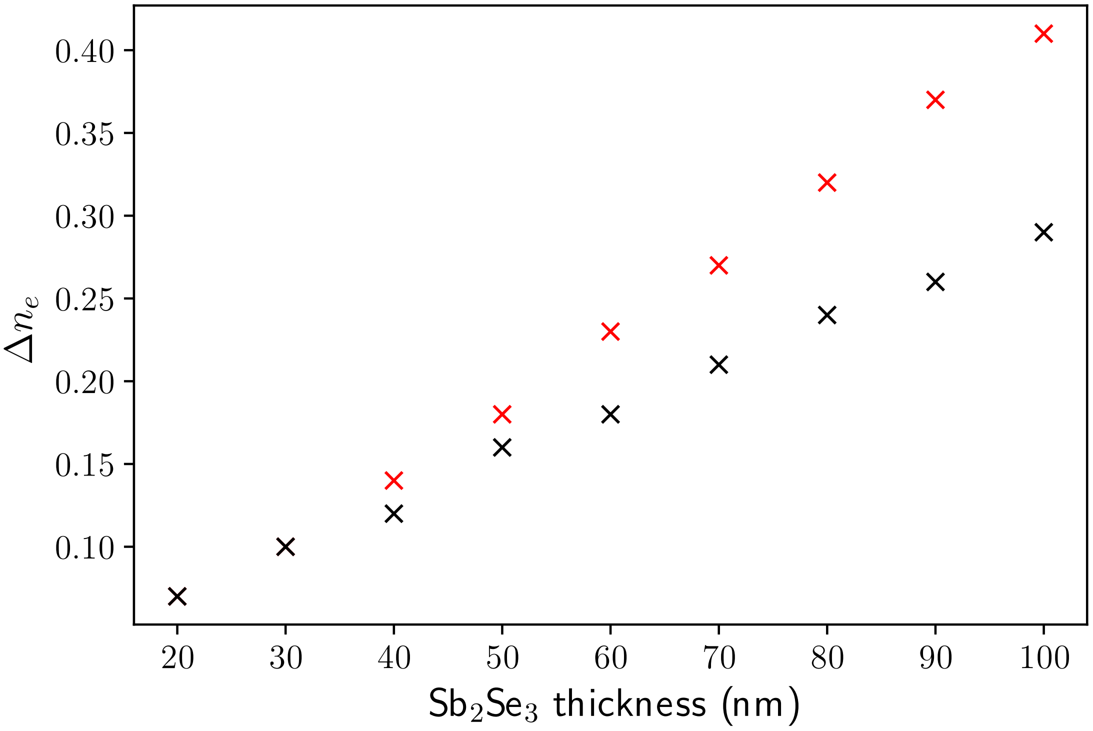

Figure S4: Cross-sections of Figure S3 showing induced change in effective refractive index n_{eff} of the fundamental mode when Sb_2Se_3 is switched from the amorphous to the crystalline states against Sb_2Se_3 thickness, when the material is deposited on the surface of the waveguide (black crosses) and when the waveguide is partially etched (red crosses).

production ready capabilities, but other lithography tools such as electron-beam lithography are frequently used in research where greater versatility of feature size is required. Rib waveguides were formed by etching 120 nm into the surface layer of silicon with a 2 μm wide trench region. Grating couplers were written with an etch depth of 70 nm and width of 1 μm, tapering down to a 500 nm width waveguide for single mode propagation. All chips contain a variety of waveguide devices, and are left with an open window following a second DUV exposure with alignment of the deposition windows to the waveguides ready for etching and deposition of Sb_2Se_3 .

2 Setup

2.1 Spectral Analysis

The experimental setup used was a conventional silicon photonics spectral analysis setup. The setup used for this experiment employed an Agilent Technologies tunable laser source with built-in detector. This light is coupled into the chip under test through the grating couplers at the inputs and outputs of a device and coupled out again to the detector via optical fibres, mounted on fixed-angle fibre arms. The chip is placed on a stage which is capable of motion in all three dimensions and viewed through a digital camera from above for fibre alignment.

In order to investigate the difference in spectral insertion loss through a variety of devices incorporating Sb_2Se_3 at a thickness of 80 nm and etch depth of 60 nm when the PCM is switched, an as-deposited amorphous sample was first tested. For every device on the chip, an insertion loss spectrum from 1520 - 1570 nm was taken, both for devices with Sb_2Se_3 embedded as well as those without for the purpose of referencing. The chip was then annealed at 190°C for 10 minutes in order to crystallize the Sb_2Se_3 areas within the devices. Then, the spectral loss measurements were repeated.

2.2 In-situ Optical Spectroscopy and Laser Writing of Phase Change Components

This experimental setup is capable of both optically switching the material and measuring C-band telecommunication wavelength transmission through the waveguides. This was used to demonstrate the switching capabilities of the novel method used in this project to embed Sb_2Se_3 into silicon photonic waveguides. Switching was performed using a Vortran 638 nm laser, digitally gated using a Berkeley Nucleonics programmable pulse generator, with light directed onto the surface of the sample via a Mitutoyo 50× objective, with NA 0.65 and focal length 4 mm. Gating the pulse using a pulse generator allows control of the pulse

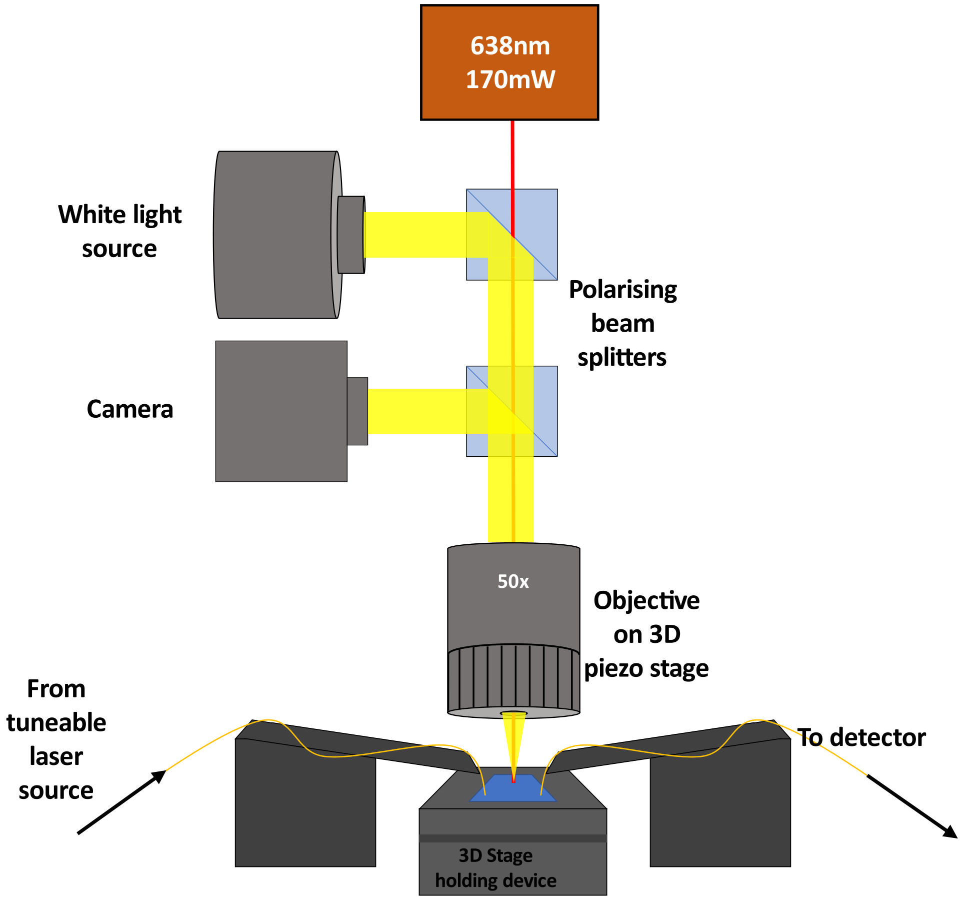

Figure S5: Optical setup used for simultaneous fibre-coupled spectroscopy and direct-write optical switching of PCMs from the top of the device under test.

down to nanosecond lengths and utilising the full 160 mW power capability of the laser in increments, enabling production of the required pulses for PCM switching. Light is coupled into the device via optical fibres from a tunable laser source to a detector, hence changes in transmission due to phase change of the material can be measured as change in transmission through the device.

To isolate the effect of changing the phase of Sb_2Se_3 integrated into a photonic waveguide, MZIs were measured. Measuring an MZI allows extraction of the phase shift from interference spectrum which is robust to variations in the coupling efficiency, for example, due to environmental fluctuations. Measuring through an interferometer provides direct self-referencing as the light travels through two waveguide arms, one clad in Sb_2Se_3 , so that the resulting interference pattern provides a measure of the phase shift incurred through one

Table S1: The pulse duration and laser power used to optically crystallize and amorphize Sb_2Se_3 of five different thicknesses deposited onto a 220nm silicon photonic waveguide.

Pulse Parameters for Optical Switching				
	Crystallization		Amorphization	
Sb_2Se_3 Thick-ness(nm)	Pulse Length (ms)	Pulse Power (mW)	Pulse Length (ns)	Amo Power (mW)
20	200	15.0	80	136
40	200	10.2	60	85.0
60	70	3.8	70	51.0
80	70	2.9	80	61.2
100	80	3.0	80	54.4

arm compared to the unclad arm.

Initially, MZIs were measured for the five different deposition thicknesses fabricated, for the six available lengths of crystalline PCM deposited on the top arm of each MZI, to investigate the losses incurred at each. Then, for a PCM length of 50µm, as-deposited amorphous Sb_2Se_3 was crystallized 1 μm at a time, with the spectrum measured after each switching event, showing the gradual phase shift possible by increasing the amount of crystalline material included in one arm. Switching between the two states was then demonstrated, beginning in an as-deposited amorphous state, crystallising 1 μm at a time and then amorphising 1 μm at a time to return to the initial state.

This experimental setup was controlled by a labVIEW program written in order to pattern the material and measure transmission through a double fibre output arm simultaneously. During the experiment a pixel pattern was written on an MMI to change the ratio of the output couplers, thus demonstrating use of Sb_2Se_3 for programmability. Each pattern was trialled in the setup, crystallizing pixels on an amorphous background and measuring the transmission through both output couplers after each pixel is crystallized. From these measurements, the change in the splitting ratio between the two output couplers can be observed.

This was then performed with a crystalline background and amorphous pixels written. Initially, an amorphous background was chosen as the refractive index of amorphous Sb_2Se_3

is closer to that of silicon than its crystalline refractive index, meaning that loss is minimized in the amorphous state. However, in practice, writing crystalline spots onto an amorphous background is less easy to control than writing amorphous spots onto a crystalline background, in part due to their larger size, and also the nature of crystals to grow when close to other crystals.

3 Fitting

Experimental results for insertion loss measurements on the MZIs were fitted using a non-linear least-squares method implemented in Python through the lmfit package.

First, spectra were converted from wavelength to frequency scale and resampled to equal frequency steps, in order to facilitate fitting using a periodic frequency comb function. To provide an initial value for fitting, the free-spectral range (FSR) of the first spectrum (before laser writing) was extracted by using a fast-fourier transform. The first spectrum was subsequently fitted to the MZI response function, resulting in values for the transmission, contrast and FSR.

For subsequent spectra, the starting value of the first FSR was used as an input for the next spectrum, where the fitting was constrained to a small variation of less than 0.1% of this frequency to take into account the shifting of the peak. The fitted FSR for each spectrum was then used as a starting point of the next spectrum in the series. This method allowed the fits to converge well even for low contrast values. Typical results are shown in S6a for the device with 20 nm Sb_2Se_3 thickness, where the red curves indicate the fits and black curves the experimental spectra. Resulting values for the transmission, contrast and FSR are given in S6b.

To complement the subset of results shown in the main text, full sets of data are presented in Fig. S7 for the five MZI devices for different Sb_2Se_3 thickness. We also performed measurements on two other chips with similar devices, yielding very similar results, results

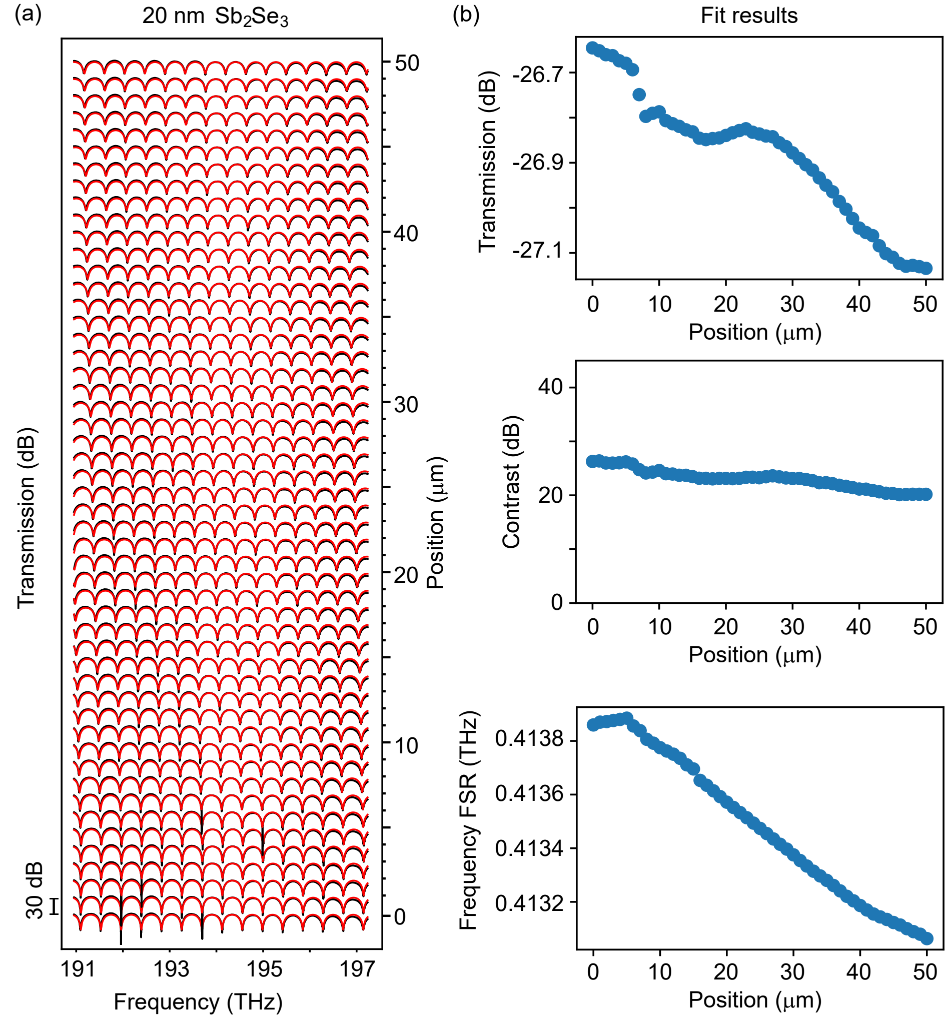

Figure S6: **Example of fitting MZI transmission spectra for 20 nm Sb_2Se_3 .** (a) All spectra corresponding to map of Fig. 2a in main text, for experimental data (black curves) and model fits (red curves). (b) Fitting results for absolute transmission, MZI extinction contrast and free spectral range (c), against position of the write laser on the PCM slab.

are shown in Fig. S8 and Fig. S9. In these two chips, a thin SiO_2 buffer layer was deposited between the Si waveguide and the PCM. The similarity of results indicate that the oxide buffer layer has no measurable effect on the performance of the hybrid PCM-Si photonics device.

In the presentation of our main results in Fig. 3 of the main text, we have chosen to show wavelength shift at a selected wavelength to illustrate the effect of the switching of the

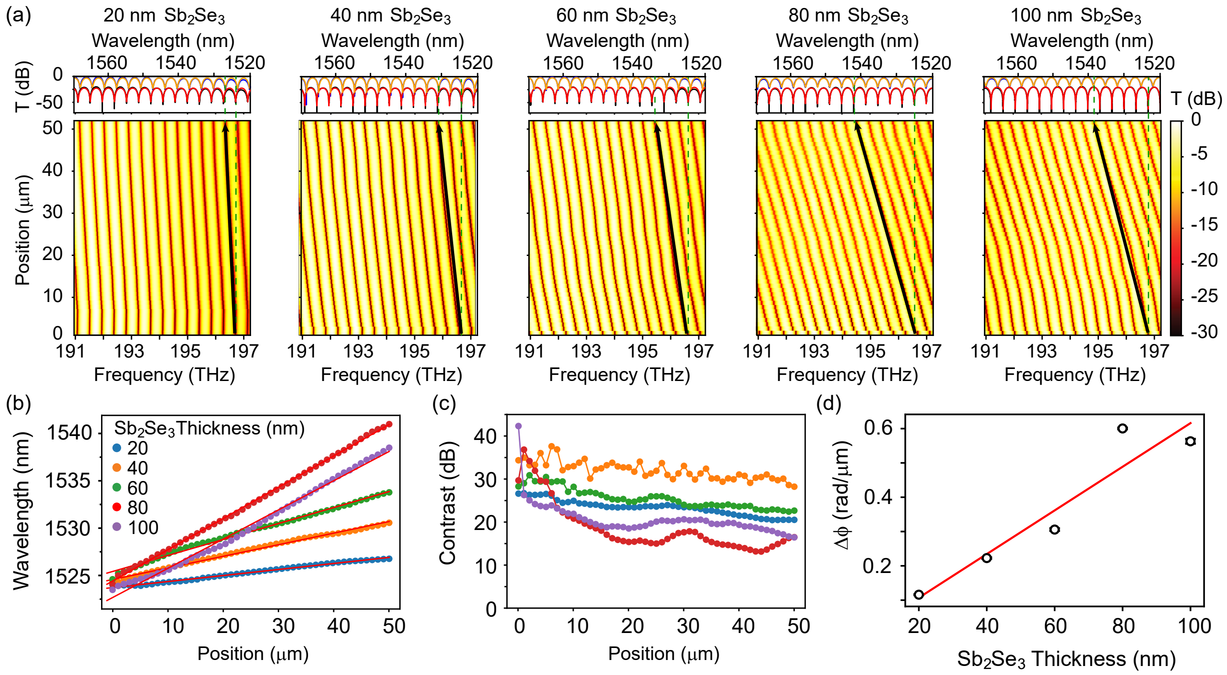

Figure S7: **Results on chip without oxide buffer layer.** (a) Spectra of MZIs before and after crystallization (top panels) and maps taken after every 1μm of crystallization (bottom panels) by direct laser writing over 50μm of Sb_2Se_3 , for devices with Sb_2Se_3 thickness of 20, 40, 60, 80, 100 nm. Black/blue curves in top panel: experimental spectra, red/orange curves: model fits. Vertical dashed lines indicate shift of selected mode with initial wavelength around 1524 nm. (b, c) Wavelength position (b) and contrast (c) extracted from fits to experimental spectra for MZI devices with Sb_2Se_3 thickness between 20 nm and 100 nm. Red lines: linear fits to data. (d) Extracted values of slope $\Delta\phi$ plotted against Sb_2Se_3 thickness. Red line: linear fit.

PCM on the spectral response. The wavelength shift was then used in combination with the FSR wavelength in this part of the spectrum, to extract the phase shift $\Delta\phi$.

In a more formal analysis one can express the frequency shift Δf in terms of the change of the FSR frequency itself, Δf_{FSR}, times the mode number N, $\Delta f = N\Delta f_{FSR}$. For the example in Fig. S6, the mode at frequency 196.6 THz corresponds to $N = 475$ given the FSR of $f_{FSR} = 0.41385$ THz. In Fig. S10 we plot the normalized f_{FSR} against length of the switched PCM for the different Sb_2Se_3 thickness. We see a change in FSR over the switched device length of up to 1.2% for the 80 nm thick Sb_2Se_3 layer, compared to a change of around 0.1% for the 20 nm Sb_2Se_3 layer. The phase shift per unit length $\Delta\phi$ then follows as $\Delta\phi = 2\pi\Delta f_{\mathrm{FSR}}/L$ resulting again in Fig. S7d or Fig. 3d in the main text.

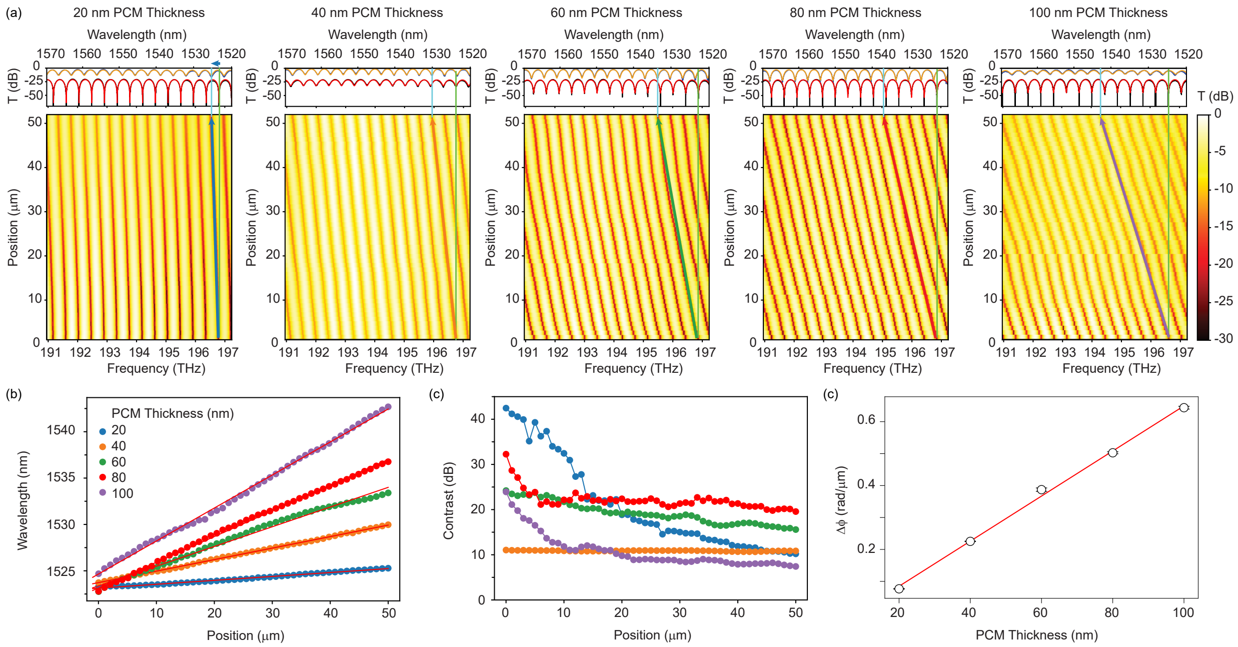

Figure S8: **Results on chip with oxide buffer layer, set 1.** (a) Spectra of MZIs before and after crystallization (top panels) and maps taken after every 1μm of crystallization (bottom panels) by direct laser writing over 50μm of Sb_2Se_3 , for devices with Sb_2Se_3 thickness of 20, 40, 60, 80, 100 nm. Black/blue curves in top panel: experimental spectra, red/orange curves: model fits. Vertical dashed lines indicate shift of selected mode with initial wavelength around 1524 nm. (b, c) Wavelength position (b) and contrast (c) extracted from fits to experimental spectra for MZI devices with Sb_2Se_3 thickness between 20 nm and 100 nm. Red lines: linear fits to data. (d) Extracted values of slope $\Delta\phi$ plotted against Sb_2Se_3 thickness. Red line: linear fit.

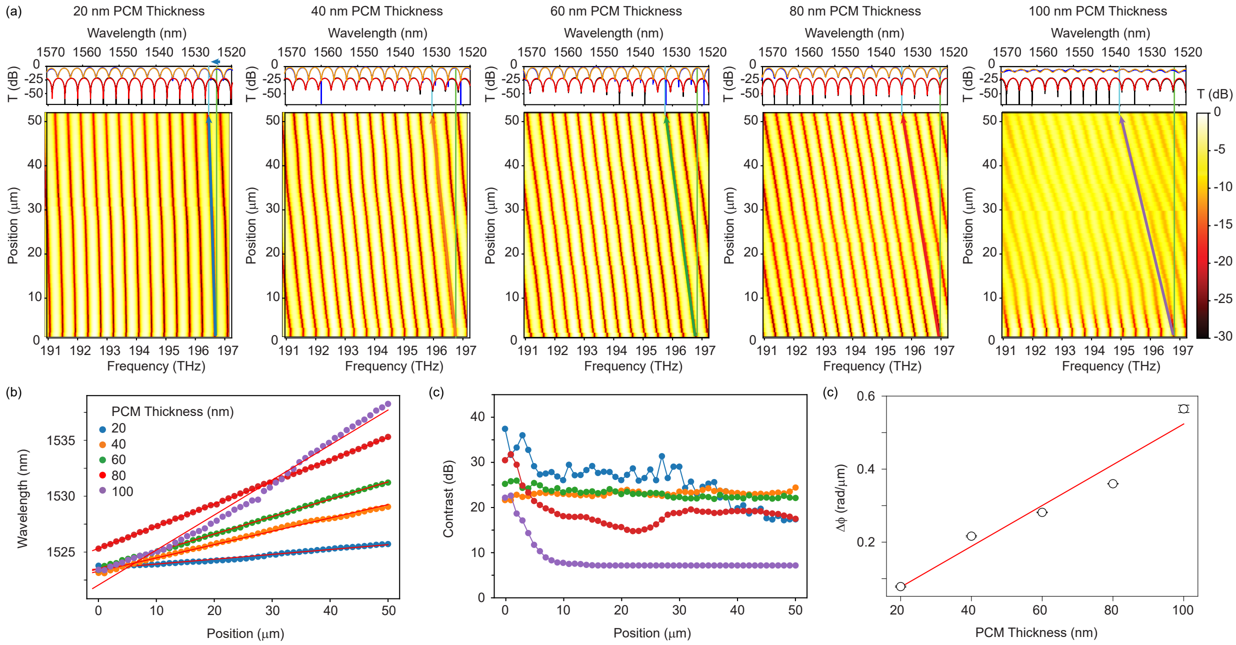

Figure S9: **Results on chip with oxide buffer layer, set 2.** (a) Spectra of MZIs before and after crystallization (top panels) and maps taken after every 1μm of crystallization (bottom panels) by direct laser writing over 50μm of Sb_2Se_3 , for devices with Sb_2Se_3 thickness of 20, 40, 60, 80, 100 nm. Black/blue curves in top panel: experimental spectra, red/orange curves: model fits. Vertical dashed lines indicate shift of selected mode with initial wavelength around 1524 nm. (b, c) Wavelength position (b) and contrast (c) extracted from fits to experimental spectra for MZI devices with Sb_2Se_3 thickness between 20 nm and 100 nm. Red lines: linear fits to data. (d) Extracted values of slope $\Delta\phi$ plotted against Sb_2Se_3 thickness. Red line: linear fit.

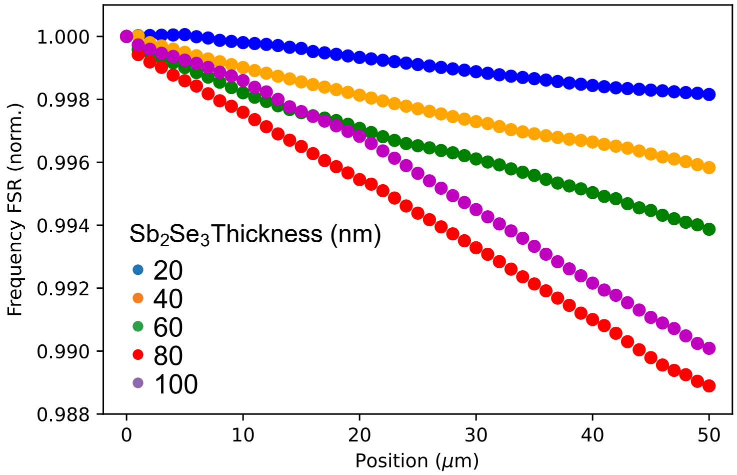

Figure S10: Fitted free spectral range normalized to initial value before switching, against write laser position along the PCM slab, for devices with different Sb_2Se_3 thickness of 20, 40, 60, 80, and 100 nm.

4 Set and reset of an MZI

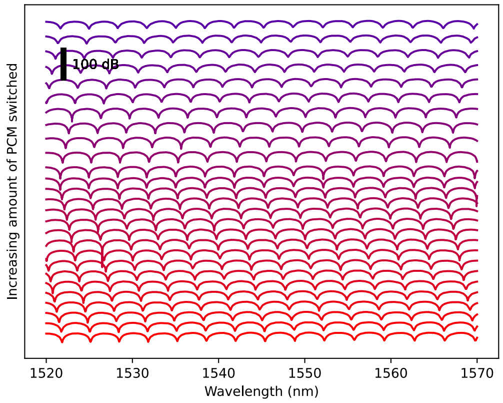

(a) 40nm deposition

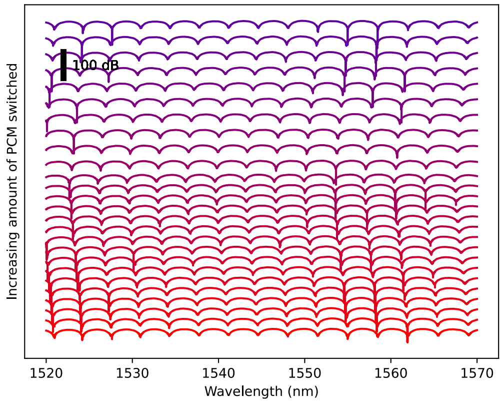

(b) 60nm deposition

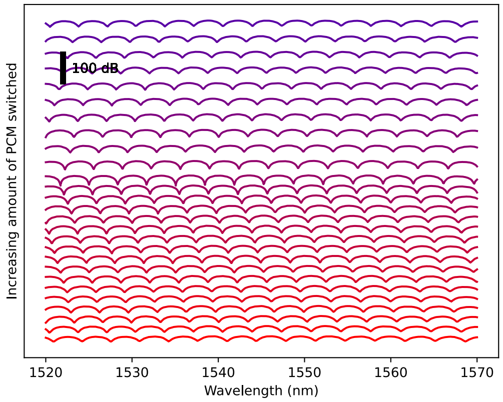

(c) 80nm deposition

Figure S11: Switching 1μm of PCM on an MZI arm and taking the spectrum after each switching performed. These Figures show every fifth spectrum taken as 50μm in total was amorphized and recrystallized, after an initial burn-in crystallisation.The subfigures show this process for three different Sb_2Se_3 thicknesses of 40 nm (a), 60n m and 80 nm.

By scanning the laser spot in steps across the device we can either set or reset pixel by pixel the Sb_2Se_3 . The step size was chosen to match the size of the device area that could addressed with a single illumination spot, and was chosen slightly larger for the crystallization than for the amorphization pulses. In our experiment we measured a full spectrum after every switching spot, which allowed us to build up a detailed picture of how the slab of PCM was switched along its length pixel by pixel. Figure S11 shows resulting spectra for for devices

with Sb_2Se_3 thickness of 40 nm, 60 nm and 80 nm. Here we show the spectra for every 5th pixel along the MMI. The wavelength shift was extracted and shown in Fig. S12. As the pre-crystallized material is gradually amorphized, the wavelength of a selected spectral peak shifts in the negative direction, and as it is gradually recrystallized, this peak shifts back in the positive direction, returning to its initial position, with an accuracy ranging from 3-20% of the initial value.

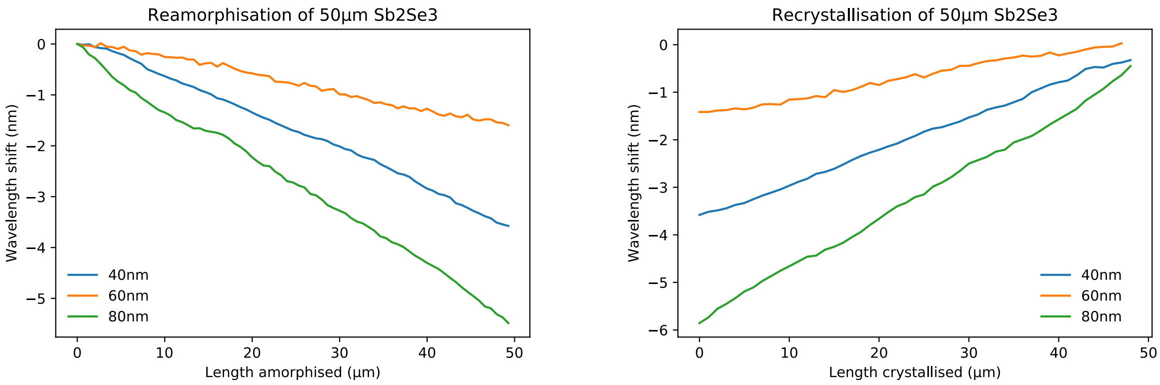

(a) (b)

Figure S12: Wavelength shift of spectra taken after each 1μm of switching is performed. This data is taken after an initial burn-in crystallisation step is performed on the as-deposited amorphous material. 50μm are then gradually (a) reamorphized and (b) recrystallized.

5 Results for switching of 60 nm Sb_2Se_3 and etch depth effect on MMI pixel patterns

In order to reduce the density of results in the main text, a subset of results were presented in Figs. 4 and 5 for Sb_2Se_3 thickness of 20, 40, 80, and 100 nm. Figure S13 summarizes the corresponding results for the 60 nm Sb_2Se_3 , both for amorphous pixels on crystalline background (top panel) and crystalline pixels on amorphous background (bottom panel). These findings are in line with the other results and lie in between those of the 40 nm and 80 nm thick Sb_2Se_3 layers in terms of number of pixels.

The figure also shows simulations where we explored a range of etch depths between 0% and 100% of the designed etch depth. This variation takes into account uncertainty in the experimental device cross section as discussed in Fig. 1 of the main text which could contribute to some of the deviation seen in experiments, in addition to the reduced pixel size. This simulation study also provides some information regarding the robustness of the design solutions against variations in device geometry.

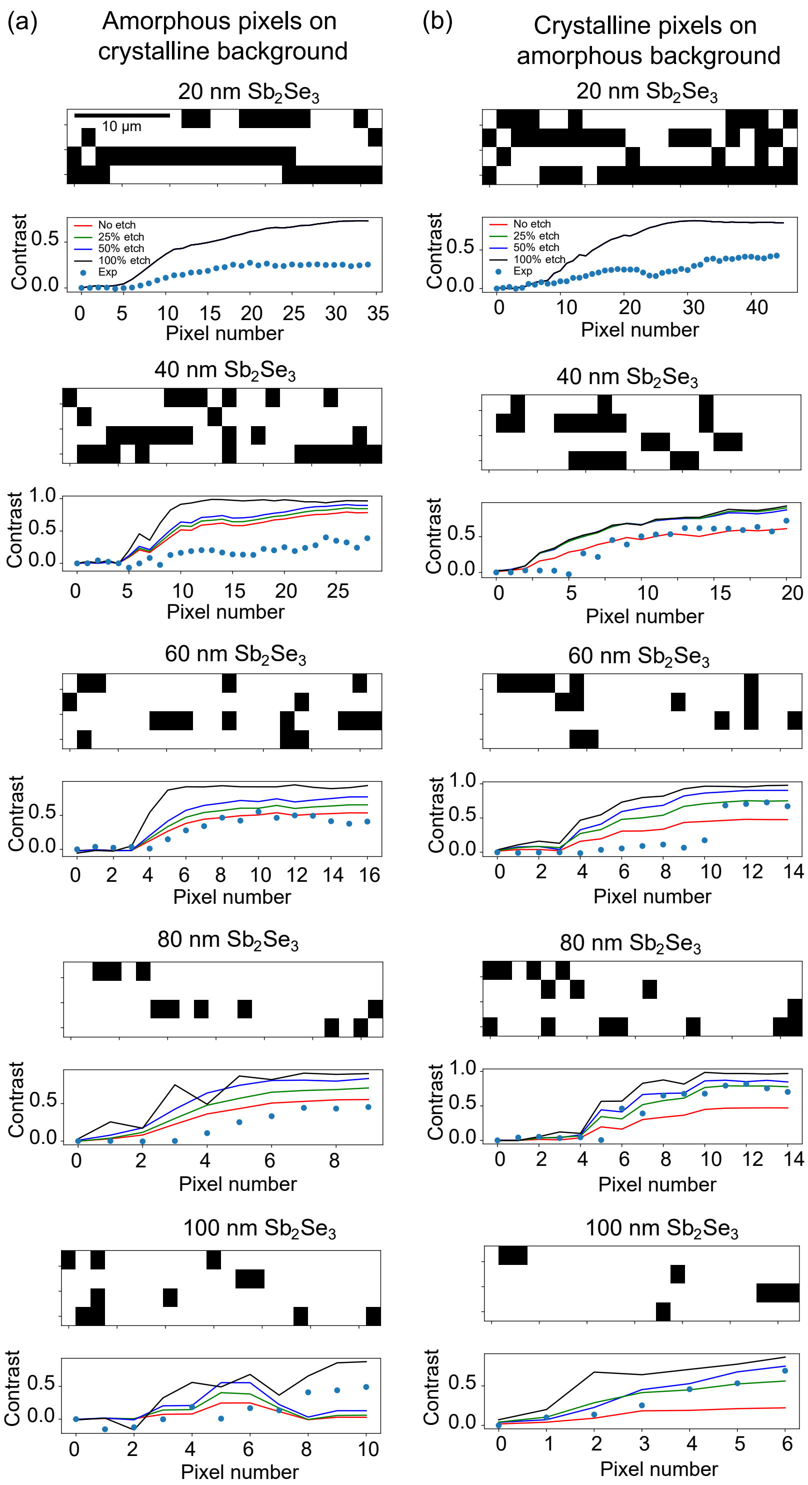

Figure S13: Switching contrast against pixel number for the digital patterns for MMI devices with 20, 40, 60, 80, and 100 nm Sb_2Se_3 thickness, with simulations performed at different etch depth conditions of 0% (no etch), 25%, 50% and 100% of the Sb_2Se_3 layer thickness minus 20 nm, and experimental results (blue dots). Data presented for amorphous pixels on crystalline background (a) and crystalline pixels on amorphous background (b).

References

(1) Xu, C. L.; Huang, W. P. Finite-Difference Beam Propagation Method for Guide-Wave optics. *Progress in Electromagnetics Research* **1995**, *11*, 1–49.

(2) Kane Yee Numerical solution of initial boundary value problems involving maxwell's equations in isotropic media. *IEEE Transactions on Antennas and Propagation* **1966**, *14*, 302–307.

(3) Yang, S.; Ma, Y.; Hochberg, M.; Baehr-Jones, T.; Novack, A.; Ding, R.; Lim, A. E.-J.; Lo, G.-Q.; Zhang, Y. Ultralow loss single layer submicron silicon waveguide crossing for SOI optical interconnect. *Optics Express, Vol. 21, Issue 24, pp. 29374-29382* **2013**, *21*, 29374–29382.
